# Application of Filter Bank to Improve Fatigue Monitoring in Wearable EEG-Based Brain–Computer Interface

**DOI:** 10.3390/neurosci7030064

**Published:** 2026-05-30

**Authors:** Timothy Jern Yu Tan, Zhuo Zhang, Kai Keng Ang, Jennifer Ang

**Affiliations:** 1 School of Chemistry, Chemical Engineering and Biotechnology (CCEB), Nanyang Technological University, 50 Nanyang Avenue, Singapore 639798, Singapore; ttan093@e.ntu.edu.sg; 2 Institute for Infocomm Research, Agency for Science, Technology and Research (A*STAR), 1 Fusionopolis Way, #21-01 Connexis (South Tower), Singapore 138632, Singapore; zhang_zhuo@a-star.edu.sg; 3 College of Computing and Data Science, Nanyang Technological University, 50 Nanyang Avenue, Singapore 639798, Singapore; 4 Home Team Science and Technology Agency (HTX), 1 Stars Avenue, #12-01, Singapore 138507, Singapore; jennifer_ang@htx.gov.sg

**Keywords:** electroencephalography, brain–computer interface, filter bank

## Abstract

Fatigue monitoring and detection are crucial for improving efficiency and safety due to their influence on reducing cognitive and physical performance that may result in safety-related incidents. This paper proposes a filter bank-based approach that decomposes electroencephalography (EEG) signals into delta, theta, alpha, beta, and gamma sub-bands for feature extraction to enhance fatigue detection using a wearable EEG-based brain–computer interface (BCI). The study utilized a publicly available EEG dataset from 40 participants collected with a dry-EEG headband while performing two cognitive tasks: a Cognitive Vigilance Task (CVT) and a Multi-Modal Integration Task (MMIT). The data was previously investigated for stress detection on the MMIT. In this study, we investigate fatigue detection on the CVT. Subjects who were not fatigued post-CVT were iteratively removed. Two models were trained with five models to classify the fatigued state from the non-fatigued state, one using features extracted from a broadband filter approach and the other from the proposed filter bank approach. Leave-one-subject-out cross-validation yielded accuracies of 75.8% ± 10.4% (95% confidence interval) from the broadband filter approach, and 86.4% ± 8.3% (95% confidence interval) from the proposed filter bank approach, yielding an overall increase of 10.6%. These results demonstrate the potential of filter bank-based feature extraction for fatigue detection in wearable EEG-based BCI systems.

## 1. Introduction

Fatigue and drowsiness are two of the largest factors that reduce performance efficiency. This introduces risks in healthcare roles where performance directly impacts the lives of others [1]. Furthermore, fatigue poses a danger in workplaces with inherent hazards such as those in the aviation, nuclear power, and mining industries [2,3]. Most notably, fatigue plays a major role in road-related incidents that occur in the early hours of the day [4]. However, the build-up of fatigue occurs at a different rate for different people, and existing policies might not mitigate the fatigue-associated risks for some people [5]. Hence, being able to monitor fatigue levels directly is essential for safety.

Proactive fatigue detection methods have been summarized [6] into three main approaches: (1) behavioral models that rely on physically observable features such as facial features; (2) physiological methods that utilize biological signals from the body; and (3) vehicle-based models that utilize information from the vehicle itself [7,8]. In the context of a real-time fatigue-monitoring brain–computer interface (BCI), physiological methods are found to be more ideal than the other two methods as they are able to detect fatigue before any visible symptoms appear [9]. These models typically utilize electroencephalograms (EEGs), electrocardiograms (ECGs) or a combination of the two [10]. There have also been studies that have used other forms of bioelectric signals, such as electromyograms (EMGs) and heart rate variability (HRV). One study [11] used both EEG and ECG signals, where a combination of frequency and spectral features was extracted for the EEG model and HRV features such as skewness and kurtosis were extracted for the ECG model. The models were then passed through a Light Gradient Boosting Machine machine learning framework. Another study [12] proposed a similar method, but in addition to the EEG and ECG models, it also utilized EMG methods by sensing electric signals from eye muscle movements, which indicated the blinking rate of the driver. Many physiological methods typically rely on multi-channel systems that would be inconvenient for real-time fatigue detection applications. Hence, a model that leverages the ability of user-friendly wearable smartwatches, headbands and wristbands to collect physiological data such as the user’s electrodermal activity and photoplethysmography alongside EEG signals for fatigue detection has been proposed [13]. In addition to these three main approaches, there have also been studies that monitor fatigue through the use of a photonic sensing system, which combines the use of a wearable sensor, the light from a smartphone and a neural network system to monitor fatigue [14].

EEG-based fatigue detection models in particular appear to be the most popular approach in the literature [15]. To detect fatigue from the EEG signals, many studies have utilized a variety of machine learning approaches to train models. One study filtered electro-oculography artifacts from the EEG data and classified a combination of wavelet packet transform and sample entropy features using Support Vector Machine [16]. Another study used a gradient boosting decision tree model to achieve a recognition rate of 94% [17]. There has also been much research done into deep-learning approaches. Most commonly used are the convolutional neural network (CNN) models, where several studies conducted in isolated settings were able to reach accuracy scores exceeding 95% [6,18,19]. One study that used a combined model of CNN and Extreme Gradient Boosting (XGBoost), termed CNN-XGBoost Evolutionary Learning, claimed that it was able to reach an accuracy score of 99.8% [20].

Despite the widespread use of EEG-based approaches in fatigue detection, translating EEG signals into intended motor actions remains a significant challenge. One study combined detrended fluctuation analysis and discrete wavelet packet transform for feature extraction with a customized mother wavelet utilizing event-related desynchronization potential patterns that were unique to individual subjects. This approach gave an accuracy of 85.33% with a soft margin support vector machine with the generalized radial basis function [21].

An extensive review in Brain-Controlled Vehicles and Brain-Controlled Aerial Vehicles applications that used EEG-based BCI systems to remotely control different vehicles identified several challenges, such as low signal-to-noise ratio, obstacle avoidance in emergency cases, limitation of distance in communication systems, reliability of application security, and developing systems for alerting the user when concentration drops [22]. Out of these challenges, a proactive EEG-based fatigue detection system would directly resolve the final issue of alerting users when concentration falls but would continue to face a major limitation in the form of the low signal-to-noise ratio. In response to this limitation, a novel method of combining the Common Spatial Pattern (CSP) projection with a Modified Secondary Projection of the filtered Common Spatial Pattern (MPCSP), which attained higher accuracies of 14.72% and 13.33% for offline and real-time processing in comparison with the CSP projections, was introduced [23].

This study proposes a novel filter bank approach that utilizes EEG features extracted from the frequency sub-bands denoting the delta, theta, alpha, beta, and gamma brain rhythms to monitor fatigue through wearable EEG-based BCI systems. Most EEG-based fatigue monitoring studies were found to either extract features from the broadband [16] or utilize other methods such as neural networks [6]. Hence, this approach was designed to explore the possible improvements using filter banks as compared to other methods. We also propose the use of Filter Bank Common Spatial Patterns (FBCSP) as EEG features and an iterative subject exclusion algorithm to exclude unhelpful EEG data.

The rest of the paper is as follows: Section 2 provides further background on the current literature of EEG-based fatigue induction, FBCSP and Filter Bank feature extractions as well as the chosen sub-band frequencies. Section 3 then details how the experiment and EEG collection were performed, as well as the details on EEG feature extraction, subject exclusion algorithm and the model’s performance. Section 4 presents the obtained results. Section 5 discusses the results and lists the limitations and future directions of this study. Lastly, Section 6 contains the conclusion.

## 2. Related Works

In this section, we discuss related studies on key topics such as (1) passive versus active fatigue, (2) fatigue induction methods, (3) FBCSP features, (4) filter bank feature extraction for EEG features and (5) sub-frequency bands selected for filter bank feature extraction.

### 2.1. Passive Fatigue and Active Fatigue

Different definitions of fatigue exist, but they are most commonly generalized into two types: active fatigue and passive fatigue [24]. One study described active fatigue as a result of participating in cognitively challenging or stimulating tasks such as solving complex equations or performing a surgery. Conversely, passive fatigue is described as the result of repetitive, monotonous tasks such as long-haul driving and is linked to boredom [25]. Additionally, fatigue is subjective for each person, and fatigue score scales measure fatigue differently based on task monotony and personal factors. As such, objective fatigue tests are highly variable and dependent on the context in fatigue assessments [26].

### 2.2. Inducing Fatigue

Studies on EEG-based fatigue detection BCI models have typically used three main methods to induce fatigue: laboratory cognitive tasks, simulation tasks, or sleep deprivation tasks [27]. Laboratory cognitive tasks often utilize some form of complex cognitive task over a prolonged period of time, such as the use of two different cognitive tasks, a multi-modal integration task (MMIT) and a cognitive vigilance task (CVT) [28]. There have also been studies that have used visual stimuli to induce visual fatigue, which is related to mental fatigue [29]. Certain professions such as drivers and pilots often perform repetitive tasks for long periods of time and may work into late hours of the night. Hence, simulations of these professions have also been widely used to induce fatigue [20,25,30]. Furthermore, studies that required the monitoring of high levels of fatigue have been able to induce these fatigue levels through sleep deprivation tasks where participants are restricted to a certain number of hours of sleep for a number of consecutive nights [26,27,31]. In this study, we have chosen to use the EEG data found in the publicly available dataset that contained data of participants that were required to complete MMIT and CVT cognitive tasks [28]. Data where the participants had to complete CVT cognitive tasks was used in this study. Due to the nature of this CVT cognitive task, where participants are required to answer cognitively complicated tasks, it is expected that it would induce active fatigue.

### 2.3. FBCSP Features

As mentioned previously, EEG-based fatigue detection BCI models require the extraction of features that allow for the detection of motor imagery movement patterns in EEG signals. The Common Spatial Pattern (CSP) algorithm, which effectively discriminates two classes of EEG measurements in a four-stage process, assists in achieving this. It begins with filtering the EEG data through multiple bandpass filters, spatial filtering using the CSP algorithm, followed by feature selection and classification of the CSP features using machine learning algorithms [32]. The spatial filtering using the CSP algorithm is conducted by linearly transforming the EEG data using Equation (1) as shown below. Equation (1): CSP algorithm used to conduct spatial filtering of EEG data [4].

(1) $$Z_{b,i}=W_{b}^{T}E_{b,i}$$

where $Z_{b,i}\;\epsilon \;R^{c\times t}$ refers to $E_{b,i}$ after spatial filtering is conducted; $W_{b}^{T}\;\epsilon \;R^{c\times c}$ refers to the CSP projection matrix; $E_{b,i\;}\epsilon \;R^{c\times t}$ refers to a single EEG measurement from the *b*th band-pass filter of the *i*th trial; *c* denotes the number of channels; *t* is the number of EEG samples per channel; and *T* denotes the transpose operator.

The CSP features are especially effective and a popular choice when classifying 2-class motor imagery EEG data [33]. One study [34] introduced the FBCSP algorithm, which applied the original CSP algorithm after the EEG signals had passed through a band-pass filter. Another study [35], which aimed to improve a CSP-based algorithm for identifying imagery movement patterns, studied 14 different approaches and concluded that a discriminative filter bank of CSP method using a discriminative sensitive learning vector quantification system that was classified using a soft margin support vector machine with a generalized radical basis function kernel achieved the highest accuracy.

In our study, we made use of FBCSP features as the responses by the participants in the CVT cognitive tasks involved the pressing of a button, which provided task-related information.

### 2.4. Filter Bank Feature Extraction

To overcome the issue of performance degradation due to incorrect use of filter bands when extracting CSP features, we introduced a method that consisted of utilizing an FBCSP framework that divided the frequency band of 4–40 Hz equally into nine different bands (4–8 Hz, 8–12 Hz, …, 36–40 Hz) [36]. The method proposed achieved an improvement in results when compared to other CSP feature extraction methods in both the areas of performance degradation as well as issues arising due to parameter selection. In this study, we explored a similar concept but utilized 5 distinct brain rhythms of delta, theta, alpha, beta, and gamma that can be determined in the EEG signal through specific frequency ranges.

### 2.5. Chosen Sub-Bands

Delta (0.5–4 Hz), theta (4–8 Hz), alpha (8–13 Hz), mu (8–13 Hz), beta (13–30 Hz) and gamma (25–100 Hz) are six distinct brain wave patterns that can be distinguished in their corresponding EEG frequency bands [29]. Activity in the delta, theta, and alpha bands is associated with fatigue, while delta activity occurs when people are in a deep sleep. Theta activity occurs when people are fatigued and alpha activity occurs when people are actively trying to remain alert. The beta activity is mainly related to when a person is alert. The mu rhythm is active in the motor area related to hand tasks [37] and the gamma rhythm is active in performing complex cognitive tasks [38]. The delta, theta, and alpha bands, in particular, are important indicators for drowsiness from sustained activity, with the alpha band dominating in situations that result in passive fatigue [39]. Delta and theta bands appear to dominate in tasks that require high attention that result in active fatigue, such as the CVT or similar psychomotor vigilance tasks [40,41]. Hence, we believe that the sub-bands chosen for this study would provide further insight into drowsiness and fatigue.

## 3. Materials and Methods

In this section, we (1) report on the source of the EEG dataset, (2) introduce the CVT task that is used to induce fatigue and explain the (3) features extracted as well as the (4) computational model for fatigue classification before (5) evaluating the model with statistical analysis and significance measurement. Finally, we (6) discuss the contributions of the features from each of the frequency bands in developing the model.

### 3.1. Publicly Available EEG Dataset

Previous authors had proposed a stress monitoring Brain–Computer Interface system that utilized both EEG as well as ECG data [28]. The data was collected from 40 participants between the ages of 21 and 65 with a balanced ratio of gender as well as ethnicity using an electroencephalogram (EEG) headband with dry electrodes. In the original study, a signal quality check was performed. The participants were required to undergo an MMIT and CVT. Self-reported stress levels were also recorded by the participants using the Dundee Stress State Questionnaire (DSSQ). The participants from this study had consented to further research.

For this study we use the publicly available EEG dataset collected from the participants that underwent the CVT task. Although the task was originally intended to induce stress on the participants, the DSSQ revealed that the participants experienced fatigue after the CVT task. Additionally, the CVT task has been validated to induce sufficient cognitive load [36] to incite the desired active fatigue for this study.

### 3.2. Cognitive Vigilance Task (CVT)

**Figure 1:**
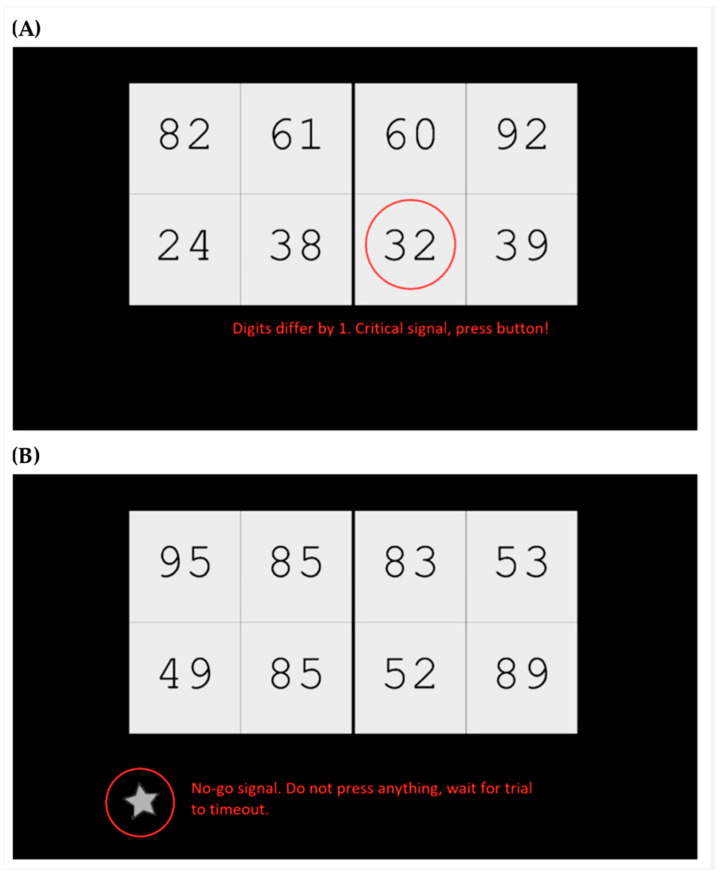
Example of the Cognitive Vigilance Task (CVT) that participants underwent. (**A**) Positive CVT trial where a number that has digits differing by a single value is present, which prompts participants to press the space bar of the keyboard. (**B**) No-Go CVT trial where a gray star is present, which prompts participants to refrain from making any action [28]. (Reprinted with permission from reference [28]. 2024, Brian Premchand).

The CVT task used in this previous study [28] consisted of a 40 min vigilance period where participants were shown multiple trials that lasted for 2.5 s each. In each trial, participants were given 8 panels of two-digit numbers, as shown below in Figure 1.

The participants were tasked with looking out for a critical condition, which occurred when at least one of the two-digit numbers had the same first and second digit or when they differed by 1, as shown in Figure 1A. If this condition occurred, it would be considered a positive trial, and they were required to press the spacebar of a keyboard. Conversely, if the critical condition was not present, it would be considered a negative trial and they would wait for the trial to time-out. Occasionally, the trial would present a no-go cue in the form of a gray star at the bottom left of the screen with at least one of the numbers meeting the critical condition, as shown in Figure 1B; in this case, the participants were instructed to let the trial time-out.

### 3.3. EEG Feature Extraction

In this study, a combination of FBCSP and EEG features was extracted in each of the frequency sub-bands of delta (2–4 Hz), theta (4–8 Hz), alpha (8–13 Hz), beta (13–30 Hz), and gamma (30–40 Hz).

As mentioned in Section 2.3, FBCSP features are typically used in motor-imagery-related EEG data. In the case of this EEG dataset, since the CVT task required the participants to either move their hand to press or withhold their hand from pressing the space bar on the keyboard, FBCSP features would be useful for this study. Furthermore, to maximize the effectiveness of the FBCSP features, the two class labels that are to be discriminated against each other should correlate to two different motor imagery actions [39].

In this experiment, the EEG data collected from the CVT task contained 8 performance codes: (100)—Blk Cue, which represented the start of the session; (120)—Critical, which indicated a positive trial; (121)—NoCritical, which indicated a negative trial; (122)—NoGoCritical, which indicated a no-go trial; (200)—True Positive, which indicated when the participant correctly answered a positive trial; (201)—True Negative, when the participant correctly answered a negative trial; (220)—False Positive; and (221)—True Negative or False Negative.

In this study, the FBCSP features extracted were CSP features taken by discriminating the performance codes of 120 and 122, which indicated a Critical and a NoGoCritical label, respectively. Since these two performance codes correlate to two distinctly different motor imagery actions of pressing the spacebar and withholding the action of pressing the spacebar, it will provide good discrimination.

The EEG features extracted are the same as the EEG features extracted in ref. [36] and included the power spectral density from each of the sub-bands. Non-linear features such as Higuchi’s Fractal Dimensions, Petrosian Fractal Dimensions, and Hjorth Parameters (Activity, Mobility and Complexity) were also extracted along with the Sample Entropy. These features have been carried forward from the previous study and used in conjunction with FBCSP features due to their repeated association with mental fatigue across various studies on fatigue monitoring [28,30]. These features assist in capturing underlying neurophysiological processes that are understood to reflect mental fatigue independent of the exact behavioral task.

A baseline set of features was also prepared by extracting the same FBCSP and EEG features using a broadband frequency across 2–40 Hz instead of from each sub-band frequency.

**Figure 2:**
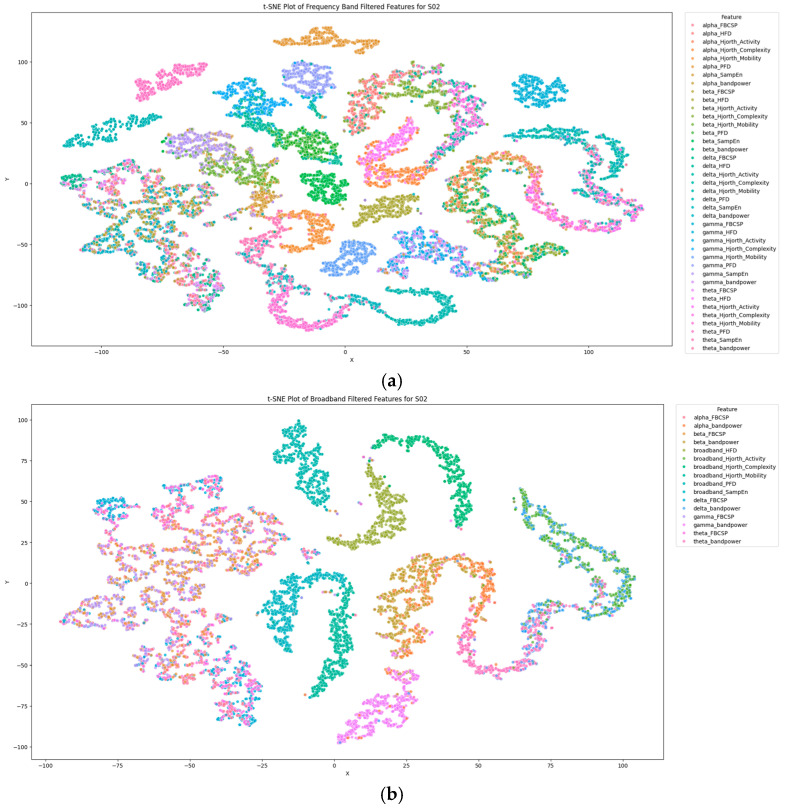
t-SNE plot for Subject 02 showing a greater variance of features in the features extracted from the sub-bands compared to the baseline. (**a**) t-SNE plot for baseline using features extracted from the broadband. (**b**) t-SNE plot for features extracted from the sub-bands.

Due to the large dimensionality of the extracted EEG features, the t-distributed Stochastic Neighbor Embedding (t-SNE) technique is used for dimensional reduction where the features extracted for each subject are mapped to a two-dimensional space while maintaining the proximity between each feature. The horizontal and vertical axes represent abstract t-SNE dimensions and are dimensionless where they do not correspond directly to any specific feature or quantity. Instead, the relative position and clustering of points represent how similar different feature patterns are. By comparing a t-SNE plot of features extracted from the broadband to a t-SNE plot of features extracted from the sub-band, it allows for better interpretation of the effectiveness of each feature by observing the clustering and variance in the plot [42]. With reference to Figure 2, it is observed that the t-SNE plot for the features extracted from the sub-band display greater variance where the features are more spread out in distinct clusters.

### 3.4. Computational Model for Fatigue Classification

**Figure 3:**
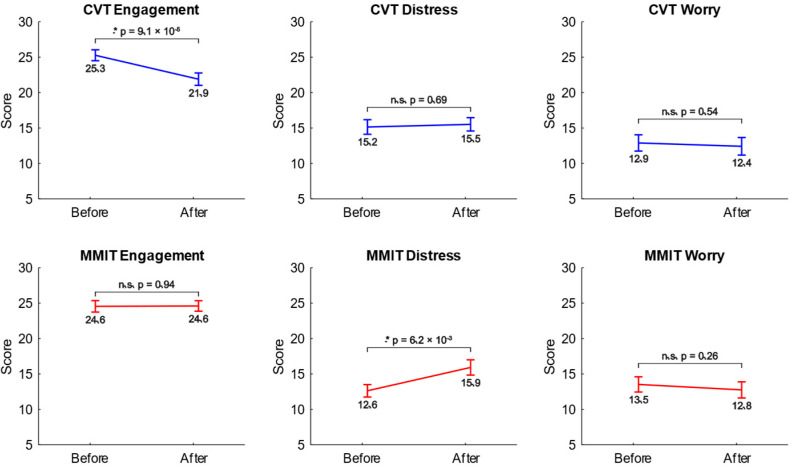
DSSQ results from previous study where n.s. *p* refers to non-significant *p*-values and * *p* refers to significant *p*-values (Reprinted with permission from reference [28]. 2024, Brian Premchand).

In the previous study [28], a laboratory cognitive task in the form of a CVT was utilized to induce fatigue on the participants. Due to the nature of this task, in which participants are required to undergo a series of cognitively complex tasks, it may have resulted in some participants experiencing excess “stress”. Despite this, it has been shown that EEG markers of mental fatigue, particularly in the delta, theta, and alpha bands, overlap with those reported for mental workload and stress, and thus any impact from additional stress would be minimal [43]. To ensure that the participants were sufficiently fatigued from the CVT tasks, self-reported levels of distress, engagement, and worry were recorded using the DSSQ, where fatigue was interpreted as the inverse of engagement. The participants filled in the DSSQ prior to and after the CVT task to record the change of mental state. Figure 3 shows the survey trends before and after participation in the CVT task. A significant drop in engagement scores post-task is shown, indicating an increase in the fatigue levels of the participants.

**Figure 4:**
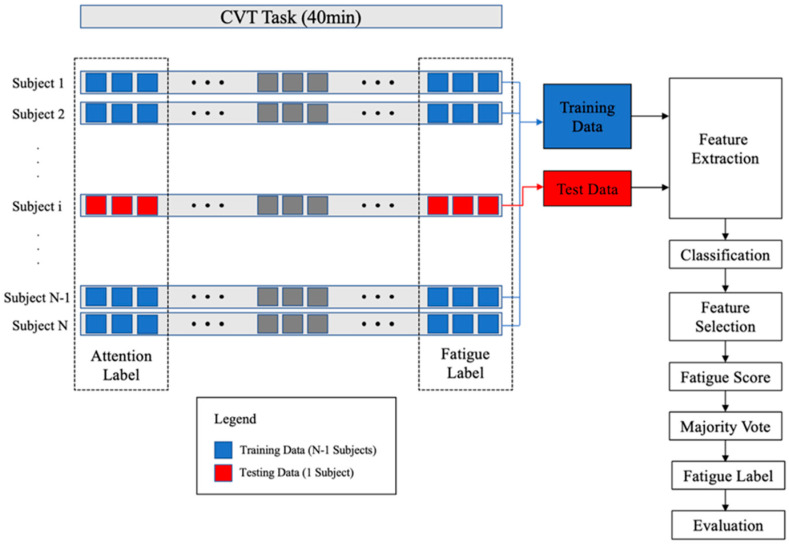
Diagram of the flow of our fatigue detection algorithm.

Additionally, in similar studies where participants have been exposed to extended fatigue inducing tasks such as an hour-long driving simulation, it was shown that there was a general increase in their fatigue score as more time passed [9], verifying that participants engaging in the CVT task would experience the most fatigue at the end of the test. To reduce non-fatigue-related noise in the EEG data, task blocks that provide the strongest indication of fatigue were identified and used for the training and evaluating of models instead of the entire dataset [28]. Hence, the first block of the first 6 epochs was labelled with a value of (0) indicating ‘alert’ and the second block of the last 6 epochs was labelled with a value of (1) for ‘fatigue’, as shown in Figure 4 below. The DSSQ results provide evidence supporting this, where the engagement decreased at the end of the CVT task, which reflects an increase in fatigue levels at the group level. However, it does not independently determine the fatigue state of each participant; hence, the ‘alert’ and ‘fatigue’ labels are more reflective of a less fatigued and more fatigued state, respectively, rather than independently verified fatigue states.

After the alert and fatigue labels were established, for each epoch, all the features were extracted on the same time window. The resulting values were flattened and concatenated into a single feature vector per epoch. These concatenated vectors were then subjected to a mutual information feature selection algorithm to filter out unhelpful features and to evaluate the performance of the model, and a leave-one-subject-out (LOSO) evaluation method was chosen to predict the scores for each of the 12 individual epochs selected. Each of these 12 scores was then compared to the mean of the scores. A majority vote was then taken in each block where the entire block was relabelled as alert (0) or fatigue (1) depending on the majority score. If there was a tie in the relabelled values of the epochs in the block, it would reflect that there is insufficient evidence to conclude that the participant is sufficiently fatigued, and the block would be labelled as alert (0).

**Figure 5:**
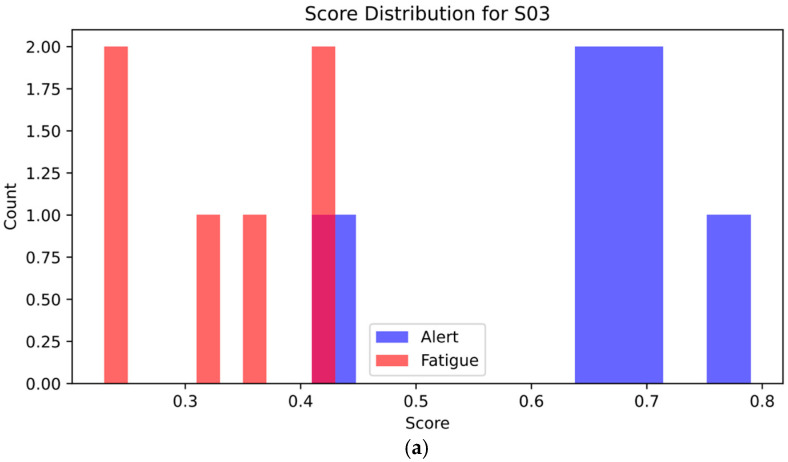

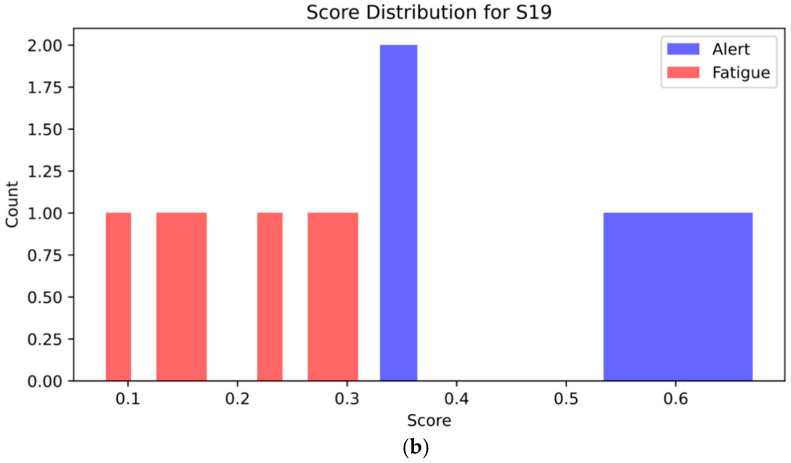
Plots of score distribution of (**a**) Subject 03 and (**b**) Subject 19 that were removed in the first and second round of Subject Exclusion, respectively. The predicted scores show the opposite of what is assumed, where early epochs predicted that the participant was fatigued and later epochs predicted the converse.

Since the DSSQ results are not reflective of individual fatigue levels, it is unlikely that all participants would begin the session being ‘alert’ and end the session being ‘fatigue’. An iterative method of subject exclusion was then implemented to remove participants whose EEG data did not follow this assumption. To accomplish this, a histogram of the predicted scores for each epoch was plotted for each participant. Since the alert block was labelled a numerical value of 0 and the fatigue block was labelled a numerical value of 1, we expected to see a low predicted value for the first 6 epochs and a high predicted value for the last 6 epochs. Hence, participants whose epoch predictions indicated the majority of the alert block to be higher than the mean and the majority of the fatigue block to be lower than the mean were identified and removed from the training and testing of the model. This resulted in the removal of subjects 03, 15 and 22, where the histogram for subject 03 can be seen below in Figure 5a.

This process was repeated again by plotting a second round of histograms using the predicted scores of the filtered subject list. This resulted in the further removal of subjects 11, 17 and 19, where the histogram for subject 19 is shown below in Figure 5b, for a total removal of 6 subjects’ data.

After the removal of all 6 subjects, the remaining subjects formed the filtered dataset, which was used for model training and evaluation. Although the final classification is still a binary decision between states of being “alert” and “fatigue”, the models used in this study produce continuous outputs that can be interpreted as fatigue scores. Specifically, regression-based models such as Random Forest Regressor, Support Vector Regressor, Gradient Boosting Regressor, K-NN Regressor and Logistic Regression were trained using the binary labels of the first and last blocks (0 for alert and 1 for fatigue), such that the predicted output represents a continuous estimate of fatigue likelihood.

These continuous outputs were subsequently used to relabel the blocks into the binary class labels using an automatic thresholding scheme based on the distribution of predicted scores, rather than manual tuning. This approach allows the models to better account for differences in EEG patterns between the individual participants while still maintaining a consistent rule to make classification decisions. In this sense, the regression models function as continuous scoring classifiers, similar to the probabilistic outputs in standard classification models.

### 3.5. Statistical Analysis

Similar to the process of the iterative subject exclusion, the epochs were evaluated under the LOSO method, where for each iteration, data from one subject was held out as the test set, while the remaining data was used for training. From the model, the overall accuracy was calculated from the confusion matrix of predicted versus true labels on the test data.

To quantify uncertainty in the accuracy scores, a 95% confidence interval was calculated based on a normal approximation to the binomial distribution of correct predictions. This approach estimates the precision of the accuracy score by considering the total number of test samples.

### 3.6. Frequency Band Contribution

**Figure 6:**
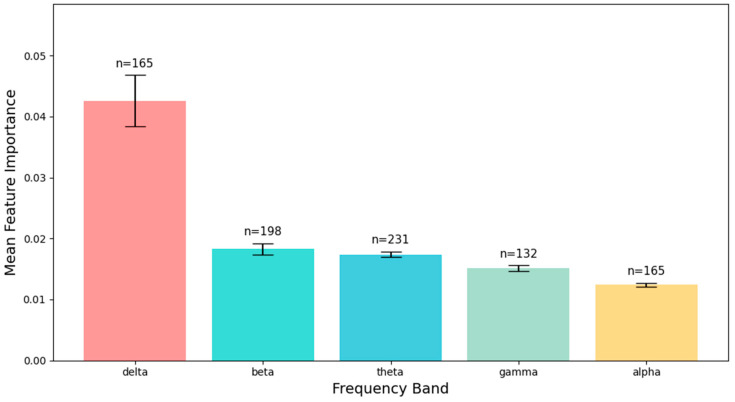
Plot showing the distribution of Frequency Band Contribution to the Random Forest Regress.

After the statistical analysis was completed, the features used were then analyzed to obtain the frequency band that was the most significant in training the model. The feature importance scores of the random forest regressor were calculated by finding the mean decrease in impurity, which was then aggregated by the frequency band. The mean importance across all features within each band was computed per subject, then averaged across the subjects to determine each band’s overall contribution to fatigue classification, as shown in Figure 6.

As can be seen in Figure 6, the delta frequency band had a significantly larger contribution of the model compared to the other frequency bands, which is validated by the studies mentioned in Section 2.5 that agree that the delta band is an important indicator of fatigue [39,40,41].

## 4. Results

Prior to the subject exclusion, the random forest model trained using LOSO evaluation yielded accuracies of 65.4% ±10.6% (95% confidence interval) from the broadband approach and 69.2% ±10.2% (95% confidence interval) from the proposed filter bank approach. After filtering the dataset, the broadband filter approach and the filter bank approach reached accuracies of 75.8% ± 10.4% (95% confidence interval) and 86.4% ± 8.3% (95% confidence interval), respectively. Thus, with the filtered dataset, the filter bank approach resulted in an overall increase of 10.6% in the accuracy score of the model. Additionally, the sensitivity and specificity of the results was 81.8% and 69.7%, respectively, for the broadband filter approach and 93.9% and 78.8%, respectively, for the filter bank approach.

## 5. Discussion

We proposed a filter-bank-based feature extraction approach to improve fatigue monitoring BCI models and evaluated it by using EEG signals from a public dataset. The public dataset contained data from 40 participants who underwent two cognitive tasks: MMIT and CVT, where only the data from the latter task was used for this study. This new approach showed a significant improvement to the accuracy scores of the model when compared with the baseline.

### 5.1. Filter-Bank

The models that were trained and tested using the filter-bank extracted features were able to achieve high levels of accuracy with the CVT training set, which aligned with the earlier observations of the t-SNE plots in Figure 2 that indicated a much wider spread and variance of features for the filter-bank extracted features as compared to the broadband extracted features. The use of a filter bank with 5 different frequency bands also means that for the same features, the filter bank method would require 5 times the computational intensity as compared to a method using features extracted from a single broadband. Hence, while this method may promote maximizing variance difference between features and provides further neurophysiological interpretation from the different frequency bands, computationally it is significantly more demanding as compared to other studies that utilize a broadband [16,17]. However, despite the fivefold increase in computational complexity, models that are built on a neural network infrastructure would still be more complex [6], making the filter bank a better alternative than neural networks for portable BCI applications.

### 5.2. Filter-Bank Common Spatial Pattern (FBCSP)

Additionally, using the Critical (120) and NoGoCritical (122) performance codes to extract the FBCSP features added the additional depth of a task-related spatial–spectral feature to the extracted features that may have provided additional information on response execution, response inhibition, target detection, or task control. Furthermore, the ability of FBCSP to learn subject-specific patterns allows for greater insight for the BCI system [34].

In this study, considering the neurophysiological importance of the delta, theta, and alpha bands as EEG markers for detecting fatigue, FBCSP features would be able to provide additional insight for the models to determine fatigue. FBCSP features are also low-dimensional and can function with limited data, allowing it to be deployed in real time on wearable systems. In comparison, a review on studies adopting neural networks to classify mental disorders showed that while neural networks would be able to learn richer, more complex patterns from EEG, they required large and diverse datasets and were computationally heavy to run [44].

### 5.3. Iterative Subject Exclusion

The CVT task is a complex task that requires the participants to be under a state of vigilance for a prolonged period of time, and it is expected that the task will not impact each participant to the same extent [45]. However it appears that for some participants, the CVT task had minimal to no impact on their fatigue levels. To a certain extent, this result could be explained by the fact that the participants of the cognitive tasks were all Singaporean working adults who often work in stressful working environments [46]. As a result of this, the 40 min CVT task may not have induced a significant mental strain on them and may have even been seen as a period of reprieve from their usual schedules. The iterative exclusion of subjects introduces potential bias and challenges the generalizability of the findings, and we acknowledge its implications for real-world deployment.

### 5.4. Limitations and Future Directions

The results obtained demonstrated that a filter-bank approach using sub-bands of delta, theta, alpha, beta and gamma is able to successfully improve the accuracy score of a fatigue monitoring model in a wearable EEG-based BCI system. However, there are still several limitations in the research that would allow for improvements in future studies.

One limitation would be that the CVT task that was supposed to cause fatigue in the participants may not have resulted in significant amounts of fatigue. This may have partially been due to the fact that different people would complete the same task with varying levels of fatigue [26]. However, EEG data taken from subjects performing a more complex or long-lasting task may have resulted in a more drastic difference between the fatigue state of the participant at the start and end of the task. Additionally, regardless of what task someone may be required to complete, as long as the task is artificially created and being completed in an isolated setting, it would not be able to fully mimic the fatigue that would be experienced in a more real-life situation. Furthermore, the participants, despite coming from a range of ages and ethnicities, were nonetheless still “Singaporean Working Adults”, which is a very narrow scope that could be further explored. Hence, for future studies, it would be insightful to expand the diversity of the participants by including participants of other nationalities and ages. Furthermore, the test environment could also be replicated in more ecologically valid scenarios such as having participants undergo extended driving simulations [9] or simulating the working environment of a healthcare worker.

The EEG signals were also collected using a dry electrode system consisting of four data channels. Despite a signal quality check being conducted in the previous study and the use of bandpass filters removing unnecessary artefacts, it still resulted in some noise when developing the model. By improving the hardware of the dry electrode system, it would be possible to improve data quality while maintaining the accessibility of this BCI system for real time use in a daily setting.

In this study, only the FBCSP features from discriminating against the Critical (120) and NoGoCritical (122) performance codes were extracted. As mentioned in Section 5.2, these features provided information regarding the differences in each trial but did not necessarily reflect information about the fatigue states of the participants itself. For future studies, different FBCSP features could be extracted by discriminating against a different combination of the performance codes that more closely reflect information on the fatigue states of the participants. Additionally, comparison of the model’s performance when using non-CSP features alone, FBCSP features alone, and the combined feature set would provide further information on the FBCSP features’ contributions to the fatigue-related classification.

As a result of variable fatigue scores due to the participant’s subjective experiences of fatigue [26], an iterative subject exclusion algorithm was employed to remove the EEG data from participants who did not experience higher levels of fatigue at the end of the CVT task. However, this might introduce a selection bias to the results obtained due to the exclusion being based on iterative predicted outcomes. Future studies could thus look to utilizing more subjective test methodologies to measure fatigue to remove this variability.

Although our combined FBCSP and filter-bank feature extraction approach attained high accuracy scores, it is also key to note that many studies have been moving away from machine learning models to more complex neural network models where the feature extraction step is not required [47]. With this in mind, a follow-up study could be performed to compare the accuracy scores of our proposed method with the arising neural network models using the same EEG dataset.

In addition, more effort could be placed into converting the offline analysis of this laboratory dataset into a more practical wearable real-time fatigue monitoring BCI system. Work could be done to evaluate online inference latency by optimizing models for low-latency deployment and individual calibration costs could also be reduced through subject-specific or transfer learning means. Furthermore, other areas of investigation could be cross-day and cross-scenario stability, robustness to motion artifacts, different wearing conditions, and fault tolerance to channel dropout. False positive and false negative costs can also be monitored to validate performance across multiple devices, subjects, and recording sessions, thereby demonstrating that the proposed approach can maintain reliable fatigue detection under realistic wearable EEG noise and artifact conditions.

## 6. Conclusions

This paper introduced a novel approach to improve the accuracy of EEG-based Brain–Computer Interface systems that are used for fatigue detection by utilizing a filter bank approach comprising the frequency sub-bands of delta, theta, alpha, beta and gamma to extract electroencephalography features. Experimental results showed that the proposed filter bank approach yielded an accuracy score of 86.4% ± 8.3% (95% confidence interval), whereas baseline features yielded an accuracy of 75.8% ± 10.4% (95% confidence interval), showing an overall increase of 10.6%. Hence, this approach significantly enhances the accuracy score of fatigue detection models and offers promising applications in other BCI systems.

## Data Availability

The data from the previous study supporting the reported results can be found from reference [28].
